# Physical Activity of Children and Adolescents during the COVID-19 Pandemic—A Scoping Review

**DOI:** 10.3390/ijerph182111440

**Published:** 2021-10-30

**Authors:** Lea Rossi, Nick Behme, Christoph Breuer

**Affiliations:** Institute of Sport Economics and Sport Management, German Sport University Cologne, 50933 Cologne, Germany; n.behme@dshs-koeln.de (N.B.); breuer@dshs-koeln.de (C.B.)

**Keywords:** exercise, sports, lockdown, public health

## Abstract

To counteract the COVIC-19 pandemic, many governments have introduced social distancing measures. While these restrictions helped contain the virus, it had adverse effects on individuals’ mental and physical health—especially children. The aim of the present study is to review the evidence on the effects of COVID-19 restrictions on children’s physical activity and their determinants. A scoping review was conducted in the databases PubMed, Web of Science, SportDiscus, and BISp-Surf. Inclusion criteria were empirical and peer-reviewed studies, youth samples, investigation of COVID-19 restrictions, and investigating changes and/or determinants of physical activity before and during the COVID-19 pandemic. Risk of bias was assessed using the checklist by Downs and Black. The search resulted in 1672 studies, of which 84 studies were included in the analysis. The results highlighted a decrease in physical activity during the pandemic, ranging between −10.8 min/day and −91 min/day. If an increase was detected, it related to unstructured and outdoor activities. The main determinants of children’s physical activity during the pandemic were age, gender, socioeconomic background, and the outdoor environment. The results imply that governments need to consider the negative effects that restrictive measures have on children’s physical activity and act to ensure high levels of physical activity.

## 1. Introduction

Since December 2019, the SARS-CoV-2 virus, responsible for the COVID-19 disease, has spread across the globe and caused a global pandemic. The World Health Organization (WHO) declared it a public health emergency of international concern on 30 January 2020 [1]. Many national governments have introduced countermeasures to counteract the disease and avoid infections, including social distancing policies, closure of schools, shops and leisure opportunities, contact restrictions, and curfews. While these restrictions effectively slowed down the spread of the virus and contained the disease, they came with negative externalities as individuals were forced to stay home, which increased the risk of social isolation [2]. Restricting leisure opportunities such as sports also created a barrier to physical activity, while at the same time, sedentary time increased during lockdown due to more time spent at home [3]. In contrast, physical activity was widely recommended by national governments during the time to maintain a healthy lifestyle [4].

One population group that has been struck especially hard by the restrictive measures is children and adolescents. While young people are less likely to experience severe symptoms after infection [5], they were still confined through school closures and the close-down of leisure activities. This confinement could have adverse effects on children’s physical and mental health as they did not get to play with their friends, be active in their sports groups, or have regular exercise in school physical education (PE) classes. This is especially dramatic as previous studies have shown that most children and adolescents did not reach the physical activity guidelines of 60 min of PA per day recommended by the WHO even before the pandemic [6]. A further reduction of physical activity during the pandemic could have harmful effects as PA during youth is an essential determinant for future PA [7], is an antecedent for mental health [8], and helps avoid future health challenges such as obesity and cardiovascular diseases [9].

While there have been several reviews on the effects of the COVID-19-pandemic on physical activity behaviors [3,4,10,11], there is a lack of systematic evidence on the effects on children and adolescents specifically. This is especially important as children below the age of 12 are at this point unable to receive a vaccination against the virus and might face further restrictions in later waves of the virus. Thus, this study aims to (1) identify how far the restrictions have impacted the physical activity levels of children and adolescents and (2) identify which factors determine the physical activity levels. The latter enables us to derive practical implications for effective strategies to raise physical activity levels in future pandemic waves, respectively, in potential future pandemics. The research questions guiding our study are thus two-fold:

**RQ1.**  *How do the restrictions during the COVID-19 pandemic affect the physical activity behavior of children and adolescents?*

**RQ2.**  *Which individual and context-specific factors determine the physical activity behavior of children and adolescents during the COVID-19 pandemic?*

To address the research questions, a scoping review of existing studies on physical activity behaviors before and during the COVID-19 pandemic was conducted. In the following, information on the methods and main results are presented. The study concludes with practical implications based on the identified effects.

## 2. Materials and Methods

A scoping literature review was conducted on the changes in physical activity levels of children and adolescents during the COVID-19 pandemic. The approach of a scoping review was chosen as the body of evidence on physical activity and COVID-19 is rapidly growing and is rather heterogenous, which is problematic for a precise systematic review. The databases searched included PubMed, Web of Science, SportDiscus, and BISp-Surf. The last date of search was 1 July 2021. The search strategy was structured with the following search terms: sport and/or physical activity, children and/or adolescents and/or youth, COVID-19 and/or Corona. Searches were conducted both in English and in German to cover a broader range of research contexts.

Studies were included when the sample contained children and adolescents (under 18 years). This included both studies that focused exclusively on children and adolescents and population studies that included children and adolescents as a subgroup in their sample. Further, studies were included when COVID-19 restrictions were investigated. Thus, only studies published after March 2020 (when lockdown measures started in most countries) were considered in the present analysis. Another inclusion criterion was the investigation of changes and/or determinants of physical activity before and during the COVID-19 pandemic. This excluded studies that investigated physical activity as a determinant for mental or physical health outcomes. This inclusion criterion chosen as the focus was specifically on physical activity as an outcome rather than as a treatment for other outcomes. Lastly, studies were included when empirical data were presented and studies were peer-reviewed. Studies were collected by one author, and two authors independently reviewed the studies for inclusion/exclusion. In case of disagreement, a final decision on inclusion/exclusion was discussed until consensus was reached.

Studies included in the qualitative synthesis were summarized by two authors in an Excel spreadsheet and information on study characteristics and main interest results were gathered. Information was summarized on the country of interest (research context), research paradigm, study design, sample size and age of interest (of children), methods used, change of physical activity behavior, and determinants of physical activity behavior. These characteristics were considered when interpreting the results of studies.

The principal summary measure was the change of physical activity behavior, which was coded “decrease” when studies reported a reduction of frequency or duration of physical activity, “increase” when studies reported growth of frequency or duration of physical activity behavior, and “no difference” when no significant differences were detected. Determinants of physical activity during the COVID-19 pandemic were gathered when significant effects were reported. Due to the high heterogeneity in the outcome measures (i.e., how physical activity was assessed), no meta-analysis could be performed and the studies were synthesized in a narrative review. Evidence was assessed to be certain when studies had low levels of risk and the majority of studies reported similar results.

Studies were assessed on their risk of bias using the checklist by Downs and Black [12]. The checklist was slightly adapted in item 27, following previous review studies [13]. Two researchers assessed each study independently, and scores were compared in the aftermath. In case of divergence, the consensus was reached through in-depth assessment by both researchers jointly. Quality levels of studies were classified as excellent (scores 26–28), good (20–25), fair (15–19), and poor (≤14) following previous applications of the Downs and Black checklist [14].

## 3. Results

### 3.1. Study Selection

**Figure 1:**
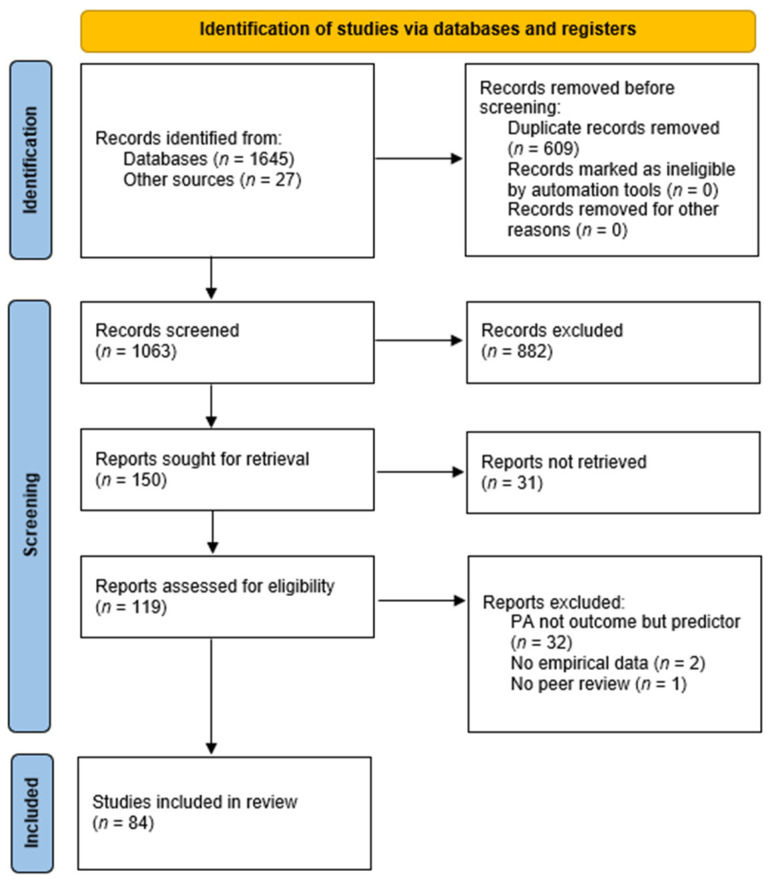
PRISMA flow chart of the study selection process.

The search and identification process is illustrated in Figure 1. 

The database search resulted in 1645 identified studies, enriched by 27 studies identified through additional searches in reference lists. After duplicates were removed, 1063 records remained, which were initially screened on eligibility based on their title and abstract. A total of 882 records were excluded as the study content and the topic of the study were considered irrelevant to the present analysis. The remaining 150 studies were sought for retrieval, of which 31 studies could not be retrieved. The remaining 119 studies were assessed on eligibility based on the full-text article, and 35 studies were excluded as they did not fulfil the inclusion criteria. Finally, 84 studies were included in the final synthesis of findings. An overview of the studies included in the analysis is presented in Appendix A.

### 3.2. Study Characteristics

The majority of studies included in the analysis stemmed from Western countries, including 25 studies from the Americas (North and South America), 33 studies from Europe, and 7 studies from Oceania. In contrast, 11 studies were conducted in Asia and only 2 studies stemmed from African countries. Five studies were classified as cross-continental as they included data from countries from different continents (see Table A1).

The research paradigm of the studies included in the analysis was predominantly quantitative, with 74 studies following a quantitative approach. Nine studies followed a qualitative paradigm and two studies used mixed-methods research. The majority of studies used a cross-sectional study design (59 studies), while 25 studies used a longitudinal design. One study employed a retrospective case-control design. The sample size of the studies included in the analyses ranged between *n* = 9 and *n* = 16,177. The total sample size cannot be stated as some studies used the same sample for their analysis. Many studies focused exclusively on children and adolescents, meaning that children and adolescents were questioned directly, or parents/caregivers were asked about children’s behavior. A total of 22 studies focused on children up to 12 years, 21 studies focused on adolescents (12–19 years), and 30 studies investigated a sample including children and adolescents (1–19 years). In the final analysis, nine studies were general population studies that included children and adolescents as subgroups. The methods used by the studies in the final analysis mainly were surveys, particularly online surveys (66 studies). Three studies conducted phone interviews, five studies used semi-structured interviews, four studies used fitness or motor competence assessments, and two studies each used accelerometery, that is, step counts. Single studies made use of a cardiopulmonary stress test, a focus group, a case study (including observations and participant journals), community mobility data via Google, and an expert panel.

The assessment of risk of bias resulted in 50 studies rated as ‘fair’, 31 studies rated as ‘poor’, and three studies rated as ‘good’. The rather low scores overall could be traced back to a lack of randomization and blinding in all studies as none of the studies were randomized-controlled trials due to the ad hoc nature of the research and the overall effect that the pandemic had on all study participants. Moreover, common concerns which occurred were the lack of a representative sample, neglection of confounders, and lack of an a priori power analysis. 

### 3.3. Change of Physical Activity Behavior

On the primary variable of interest, most studies (57 studies) reported a decrease in physical activity, both in duration and frequency of physical activity (see Table 1). Four studies identified an increase in physical activity [15,16,17,18], whereas six studies could detect no significant difference in physical activity behaviors before and during the pandemic [19,20,21,22,23,24]. Eight studies reported differentiated results with indoor and organized sports and physical activity decreasing, while non-organized and outdoor sports and physical activity increased [25,26,27,28,29,30,31,32].

The decrease in time spent on physical activity ranged between −45 min/day in Chile [33] to −91 min/day in Spain [34]. However, the measurement of physical activity differed substantially across the studies, which made the reporting of exact effects difficult. Thus, this range covers only those studies reporting concrete figures. The increase in physical activity was reported to be +53 min/day in Sweden [17].

### 3.4. Determinants of Physical Activity Behavior

Thirty-four studies identified one or more determinants of the physical activity behavior of children and adolescents during the COVID-19 pandemic. Facilitating factors, meaning factors that enabled children and adolescents to be more physically active, were found on an individual level but also on a context level. Individual factors increasing physical activity of children were being male [35,36,37], following a daily routine [27,38], spending time on outdoor physical activity [35,38], taking part in online PE classes [27,38,39], more time available [40], no school [40], use of digital platforms [41], prior fitness status [28,42,43,44], and health-related quality of life [44]. Context-specific factors, i.e., factors relating to the family context, were having more than one child at home [29,33,45], higher parental education [36], perceived parental capability [35], living in a house [46], low dwelling density [46], access to parks [46], parental encouragement and support [27,47,48], parental engagement in physical activity [47], family dog ownership [47], household income [47,48], family coexistence [49], and access and size of outdoor space available [45,48,50,51] (see Table 2).

In contrast, studies also identified constraining factors, meaning factors that hindered children and adolescents from being physically active. These constraining factors were also individual or context-specific. Individual constraining factors included the child’s age [20,37,45,50,52,53], feelings of stress [54,55], feeling comfortable at home [55], having a migration background [34], lower socioeconomic status (SES) [29], change to routines [27], pre-COVID sedentary time [44], pre-COVID activity levels [51], and mood states [56]. Context-related constraints included the caregiver’s level of education [32,33,34], living in an apartment [33], parent’s age [47], parent’s marital status [20], lack of playmates [57], family conflict [36,58], no outdoor space available [55], level of parents’ stress [49], lack of supervision [57], proximity to major roads [46], restrictions from COVID-19 [40,48], club training cancellation [40], enrolment in an early education center [33], and urban environment [51,59] (see Table 3).

## 4. Discussion

When it comes to physical activity, behaviors of children and adolescents during the COVID-19 pandemic, the level of evidence is relatively strong, with a surprisingly high number of longitudinal studies. However, the review revealed a lack of methodological variety as most studies used online surveys. Moreover, there is a clear focus on Western countries (Australia, Canada, USA, Europe) with a lack of research into Asian and African countries. More research is needed in these regions (with the exception of China) to see how children and adolescents were affected by lockdown measures there.

There is clear evidence for decreased physical activity during the lockdown in the studies identified in this review, especially in organized sports, as structured sports programs and facilities were closed. This shows that public recommendations for maintaining physical activity have so far been ineffective. If an increase has been detected, it occurred in outdoor play and unstructured activities [15,26,29,30,31] or related to lower levels of national restrictions [17]. The increase in outdoor activities depends strongly on the housing environment. Thus, the pandemic and the resulting restrictions have further increased social differences between families with safe and spacious outdoor spaces and families living in dense environments.

This is also reflected in the determinants of children’s physical activity behavior. The determinants identified by the studies mainly related to the parental and family background, the outdoor environment, participation in structured programs, including having a routine, and previous physical activity experiences. The role of parental education differed depending on the context. In European studies, higher education of parents increased physical activity, whereas in studies in Latin America, higher parental, especially maternal education harmed children’s physical activity. This difference could hint at cultural differences or issues in time availability and strengthens our call for more research in different national contexts (especially Africa and Asia).

One predictor of children’s physical activity identified in the review was the socioeconomic background. This complements general physical activity findings that children and adolescents with a higher SES are generally more physically active [60]. Once again, this difference relates to the built environment that children and adolescents are situated in as they provide little to no opportunities for physical activity—especially in times of a pandemic where indoor activities and crowded areas were restricted.

Last but not least, the problem of inactivity is especially prominent in females and older children and adolescents. This is especially concerning as the teenagers’ age group had already shown decreasing activity levels before the pandemic [60]. Thus, the restrictions and lockdowns have intensified the problem even further. One reason for the steeper decline during the pandemic could be the more substantial reliance on routines and the lack of replacement programs for organized activities. A higher priority needs to be put on adolescents’ routines to maintain a healthy and active lifestyle also during a pandemic.

The findings of the review lead to clear implications for policymakers. First, policymakers and educators need to educate and engage parents and caregivers in physical activity to increase physical activity levels overall ensuring consistent movement at baseline. One approach would be to include physical literacy into pre-school and school education agendas and make physical activity as one component of a healthy lifestyle a top priority in children’s education.

Second, to create physical activity routines and thus maintain physical activity levels during phases of lockdowns, policymakers need to make online PE classes mandatory if schools need to close down again. Programs on digital platforms have been found to have a positive effect as well but to sustain participation and ensure that routines are kept, making these programs mandatory is critical.

Third, policymakers and city planners need to ensure a safe and movement-friendly outdoor environment with access to parks and playgrounds and low-density architecture. This is needed, especially in socially deprived areas, to decrease the divide between families with higher and lower SES. This divide has been illustrated once more during the pandemic—both in infection rates and physical activity behaviors. 

Some limitations might constrain the explanatory power of this review. First, it is hard to compare all studies identified in the database search as studies were conducted during different stages of the COVID-19 pandemic. The search period included studies conducted between March 2020 and April 2021. As infection rates and restrictions differed between countries and stages of the pandemic, these findings cannot be related to specific lockdown measures. However, we tried to take these differences into account by considering the national context in interpreting studies. Moreover, the variability in measurement of physical activity across the studies limits the comparability further and makes general statements on the impact of the pandemic difficult.

Future research is needed to establish ongoing monitoring of the physical activity of children and adolescents. Potential long-term effects of the COVID-19 restrictions need to be identified, and effective countermeasures need to be developed. This review has given the first insight into the effects on children’s physical activity and what can be done to counteract the decreasing activity. However, more research is needed to assess this in the long term. Moreover, generalized tools to measure the physical activity of children and adolescents need to be established to ensure comparability of results across studies and national contexts, which again enables researchers to track global long-term changes in physical activity. Lastly, while this review has focused on the changes and determinants of physical activity, governmental restrictions and the resulting decrease in physical activity have potential further effects on children and adolescents, i.e., decreasing scholastic performance or mental health. Future reviews should focus on these outcomes of decreasing physical activity to create a more holistic picture of the effects of the COVID-19 pandemic on children and adolescents.

## 5. Conclusions

This scoping review has shown strong evidence for a negative effect of COVID-19 restrictions on children’s physical activity behavior. Physical activity has decreased especially with higher age of children and with a lower socioeconomic background. Thus, the COVID-19 pandemic has worsened the trend of inactivity which was alarming even before the pandemic.

While the review has uncovered a strong growth of research on the topic of physical activity of children and adolescents and COVID-19 since the start of the pandemic, it has also highlighted a lack of consistent measurement of physical activity levels which hinders the comparison of results between studies. Developing consistent measurement standards of physical activity should be part of a future research agenda to ensure that long-term developments of physical activity behaviors can be tracked and compared across national and international studies.

## Figures and Tables

**Table 1:** Changes in physical activity (PA) of children and adolescents during the COVID-19 pandemic.

Change of PA Identified	Min	Max	No. of Studies
Decrease	−45 min/day [33]	−91 min/day [34]	57
Increase	-	+53 min/day [17]	4
No change	-	-	6
Mixed results	-	-	8

**Table 2:** Facilitating factors in children’s physical activity (PA) during the COVID-19 pandemic.

Level	Facilitating Factor	No. of Studies
Individual level	Prior fitness status	4
	Male gender	3
	Taking part in online PE classes	3
	Following a daily routine	2
	Spending time on outdoor PA	2
	More time available	1
	No school	1
	Use of digital platforms	1
	Health-related quality of life	1
Context level	Size of outdoor space available	4
	More than one child	3
	Parental encouragement and support	3
	Household income	2
	Higher parental education	1
	Perceived parental capability	1
	Living in a house	1
	Low dwelling density	1
	Access to parks	1
	Parental engagement in PA	1
	Family dog ownership	1
	Family coexistence	1

**Table 3:** Constraining factors in children’s physical activity (PA) during the COVID-19 pandemic.

Level	Constraining Factor	No. of Studies
Individual level	Child’s age	6
	Feelings of stress	2
	Feeling comfortable at home	1
	Having a migration background	1
	Lower socioeconomic status	1
	Change to routines	1
	Pre-COVID sedentary time	1
	Pre-COVID activity levels	1
	Mood states	1
Context level	Caregiver’s level of education	3
	Family conflict	2
	Restrictions from COVID-19	2
	Urban environment	2
	Living in an apartment	1
	Parent’s age	1
	Parent’s marital status	1
	Lack of playmates	1
	No outdoor space available	1
	Level of parent’s stress	1
	Lack of supervision	1
	Proximity to major roads	1
	Club training cancellation	1
	Enrolment in an early education center	1

## Appendix A

**Table A1:** Overview of studies included in the systematic review by continent (in alphabetic order).

No.	Author, Year (Ref)	Country	Research Paradigm	Study Design	Sample (Age Incl.)	Method	Change of PA	Risk of Bias
Americas								
1	Aguilar-Farias et al., 2020 [33]	Chile	QUAN	Cross-sectional	*n* = 3157 (1–5 years)	Online survey	Decrease	Fair
2	Bazett-Jones et al., 2020 [61]	USA	QUAN	Cross-sectional	*n* = 287 (9–19 years)	Online survey	Decrease	Fair
3	Beck et al., 2021 [62]	USA	QUAN	Cross-sectional	*n* = 145 (4–12 years)	Survey	Decrease	Fair
4	Dayton et al., 2020 [63]	USA	QUAN	Retrospective case-control	*n* = 20 (mean: 15.2 years)	Cardiopulmonary stress testing	Decrease	Fair
5	Gama de Matos et al., 2020 [64]	Brazil	QUAN	Cross-sectional	*n* = 426 (children, adolescents, adults, elders)	Online survey	Decrease	Fair
6	Dos Santos Cardoso de Sá, 2020 [65]	Brazil	QUAN	Cross-sectional	*n* = 816 (<12 years)	Online survey	Decrease	Poor
7	Dunton et al., 2020 [66]	USA	QUAN	Cross-sectional	*n* = 211 (5–13 years)	Online survey	Decrease	Fair
8	Eyler et al., 2021 [67]	USA	QUAN	Cross-sectional	*n* = 245 (5–12 years)	Online survey	Decrease	Fair
9	Garcia et al., 2020 [68]	USA	QUAN	Longitudinal	*n* = 9 (14–19 years)	Online survey	Decrease	Fair
10	Guerrero et al., 2020 [35]	Canada	QUAN	Cross-sectional	*n* = 1472 (5–17 years)	Online survey, decision tree	-	Fair
11	Hemphill et al., 2020 [69]	Canada	QUAN	Longitudinal	*n* = 109 (9–16 years)	Step count via Fitbit-Tracker	Decrease	Fair
12	Kracht et al., 2021 [58]	USA	QUAN	Cross-sectional	*n* = 1836 (3–6 years)	Online survey	Decrease	Fair
13	Lafave et al., 2021 [15]	Canada	QUAL	Cross-sectional	*n* = 17 (2–5 years)	Semi-structured interviews	Increase	Poor
14	Malta et al., 2021 [70]	Brazil	QUAN	Cross-sectional	*n* = 6470 (12–17 years)	Online survey	Decrease	Poor
15	McCormack et al., 2020 [25]	Canada	QUAN	Cross-sectional	*n* = 328 (school-aged)	Online survey	PA at home: increase PA outdoors: decrease	Fair
16	Mitra et al., 2020 [46]	Canada	QUAN	Cross-sectional	*n* = 1472 (5–17 years)	Online survey	Decrease	Fair
17	Moore et al., 2020 [47]	Canada	QUAN	Cross-sectional	*n* = 1472 (5–17 years)	Online survey	Decrease	Fair
18	Pavlovic et al., 2021 [71]	USA	QUAN	Cross-sectional	*n* = 2440 (PE teachers, school and district administrators, nurses, others)	Online survey	Decrease	Poor
19	Pelletier et al., 2021 [26]	Canada	QUAL	Cross-sectional	*n* = 21 (7–12 years)	Semi-structured interview	Increase in unstructured activity; decrease in structured activity	Poor
20	Perez et al., 2021 [48]	USA	QUAL	Cross-sectional	*n* = 321 (mean: 7.8 years)	Open-ended survey	Decrease	Poor
21	Riazi et al., 2021 [16]	Canada	QUAL	Cross-sectional	*n* = 29 (5–11 years)	Semi-structured interviews	Decrease	Poor
22	Shepherd et al., 2021 [27]	Canada	QUAL	Cross-sectional	*n* = 20 (high-school student-athletes)	Semi-structured interviews	Variations	Poor
23	Siegle et al., 2020 [50]	Brazil	QUAN	Cross-sectional	*n* = 816 (<13 years)	Online survey	Decrease	Fair
24	Tulchin-Francis et al., 2021 [52]	USA	QUAN	Cross-sectional	*n* = 1310 (3–18 years)	Online survey	Decrease	Fair
25	Wahl-Alexander and Camic, 2021 [72]	USA	QUAN	Longitudinal	*n* = 264 (mean: 9.6 years)	In-person testing (BMI, fitness assessment)	Decrease	Poor
Europe								
26	Androutsos et al., 2021 [73]	Greece	QUAN	Cross-sectional	*n* = 397 (2–18 years)	Online survey	Decrease	Fair
27	Bronikowska et al., 2021 [28]	Poland	QUAN	Longitudinal	*n* = 127 (mean: 15.4 years)	Online survey	Increase in previously inactive, decrease in previously active	Fair
28	Chambonniere et al., 2021 [51]	France	QUAN	Cross-sectional	*n* = 6491 (6–17 years)	Online survey	Decrease	Poor
29	Chen et al., 2021 [74]	Sweden	QUAN	Longitudinal	*n* = 1316 (mean: 13.6 years at baseline)	Online survey	Decrease	Poor
30	Dauty et al., 2020 [75]	France	QUAN	Longitudinal	*n* = 19 (13–15 years)	Pre- and post-confinement Yo-Yo Test	Decrease	Fair
31	Delisle Nyström et al., 2020 [17]	Sweden	QUAN	Longitudinal	*n* = 100 (3–5 years)	Phone interview, accelerometer	Increase	Good
32	Di Renzo et al., 2020 [19]	Italy	QUAN	Cross-sectional	*n* = 3533 (≥12 years)	Online survey	No difference	Fair
33	Dragun et al., 2020 [20]	Croatia	QUAN	Longitudinal	*n* = 1326 (adolescents, medical students)	Pre-COVID: in-person survey During COVID: online survey	No difference	Fair
34	Fillon et al., 2021 [76]	France	QUAN	Longitudinal	n/a	Expert panel	Decrease	Poor
35	Francisco et al., 2020 [77]	Italy, Spain, Portugal	QUAN	Cross-sectional	*n* = 1480 (3–18 years)	Online survey	Decrease	Poor
36	Gallucio et al., 2021 [21]	Italy	QUAN	Cross-sectional	*n* = 91 (15–17 years)	Online survey	No difference	Fair
37	Gilic et al., 2020 [36]	Bosnia and Herzegovina	QUAN	Longitudinal	*n* = 688 (15–18 years)	Online survey	Decrease	Fair
38	Jurak et al., 2021 [78]	Slovenia	QUAN	Longitudinal	*n* = 20,000 (6–14 years)	SLOfit barometer, fitness measurements	Decrease	Poor
39	Kolota and Glabska, 2021 [22]	Poland	QUAN	Cross-sectional	*n* = 1334 (10–16 years)	Online survey	No difference	Fair
40	Kovacs et al., 2021 [38]	10 Euro-pean countries	QUAN	Cross-sectional	*n* = 8395 (6–18 years)	Online survey	Decrease	Poor
41	López-Bueno et al., 2020 [79]	Spain	QUAN	Cross-sectional	*n* = 860 (3–16 years)	Online survey	Decrease	Fair
42	Medrano et al., 2020 [34]	Spain	QUAN	Longitudinal	*n* = 290 (8–16 years)	Online survey	Decrease	Good
43	Ng et al., 2020 [40]	Ireland	QUAN	Cross-sectional	*n* = 1214 (12–18 years)	Online survey	Decrease	Fair
44	O’Kane et al., 2021 [23]	Ireland	MIXED	Longitudinal	*n* = 281 (12–14 years)	Survey, semi-structured interviews	No difference	Fair
45	Orgilés et al., 2020 [49]	Italy, Spain	QUAN	Cross-sectional	*n* = 1143 (3–18 years)	Online survey	Decrease	Poor
46	Pietrobelli et al., 2020 [80]	Italy	QUAN	Longitudinal	*n* = 41 (6–18 years)	Pre-COVID: in-person interview During COVID: phone interview	Decrease	Fair
47	Pombo et al., 2020 [45]	Portugal	QUAN	Cross-sectional	*n* = 2159 (<13 years)	Online survey	-	Fair
48	Pombo et al., 2021 [81]	Portugal	QUAN	Longitudinal	*n* = 114 (6–9 years)	Motor competence assessment	Decrease	Good
49	Pombo et al., 2021 [82]	Portugal	QUAN	Cross-sectional	*n* = 2159 (<13 years)	Online survey	Decrease	Fair
50	Poulain et al., 2021 [29]	Germany	QUAN	Longitudinal	*n* = 285 (1–10 years)	Online survey	Playing outdoors: increase Indoor sports: decrease	Fair
51	Schmidt et al., 2020 [30]	Germany	QUAN	Longitudinal	*n* = 1711 (4–17 years)	Pre-COVID: in-person survey During COVID: online survey	Nonorganized PA: increase Organized sports: decrease	Fair
52	Sekulic et al., 2020 [42]	Croatia	QUAN	Longitudinal	*n* = 401 (15–18 years)	Online survey	Decrease	Fair
53	Siachpazidou et al., 2021 [83]	Greece	QUAN	Cross-sectional	*n* = 482 (4–12 years)	Online survey	Decrease	Poor
54	Szabó et al., 2020 [84]	Hungary	QUAN	Cross-sectional	*n* = 840 (<85 years)	Online survey	Decrease	Fair
55	Ten Velde et al., 2021 [85]	Netherlands	QUAN	Longitudinal	Cohort A: *n* = 102 Cohort B: *n*=131 (A: 4–18 years B: 7–12 years)	Cohort A: online survey Cohort B: online survey, accelerometry	Decrease	Fair
56	Versloot et al., 2020 [86]	Netherlands	QUAN	Longitudinal	*n* = 82 (6–49 years)	Phone interview	Decrease	Poor
57	Vukovic et al., 2021 [43]	Serbia	QUAN	Cross-sectional	*n* = 450	Online survey	Decrease	Fair
58	Wunsch et al., 2021 [44]	Germany	QUAN	Longitudinal	*n* = 1711 (4–17 years)	Pre-COVID: in-person survey During COVID: online survey	Increase	Fair
59	Zorcec et al., 2020 [87]	Macedonia	QUAN	Cross-sectional	*n* = 72 (mean: 7.3 years)	Online survey	Decrease	Poor
Asia								
60	Constantini et al., 2021 [39]	Israel	QUAN	Cross-sectional	*n* = 473 (16–18 years)	Online survey	-	Fair
61	Elnaggar et al., 2020 [57]	Saudi Arabia	QUAN	Longitudinal	*n* = 63 (14–18 years)	Online survey	Decrease	Poor
62	Guo et al., 2021 [88]	China	QUAN	Cross-sectional	*n* = 10,416 (10–16 years)	Online survey	Decrease	Poor
63	Jia et al., 2020 [89]	China	QUAN	Cross-sectional	*n* = 10,082 (high school, college, graduate school students)	Online survey	Decrease	Poor
64	Xiang et al., 2020 [90]	China	QUAN	Longitudinal	*n* = 2426 (6–17 years)	Online survey	Decrease	Poor
65	Xiao et al., 2021 [37]	China	QUAN	Cross-sectional	*n* = 1680	Online survey	-	Poor
66	Yang et al., 2020 [91]	China	QUAN	Cross-sectional	*n* = 10,082 (mean: 19.8 years)	Online survey	Decrease	Poor
67	Zenic et al., 2020 [59]	Croatia	QUAN	Longitudinal	*n* = 823 (mean: 16.5 years)	Online survey	Decrease	Fair
68	Zhang et al., 2020 [56]	China	QUAN	Cross-sectional	*n* = 9979 (9–14 years)	Online survey	Decrease	Fair
69	Zhou et al., 2021 [92]	China	QUAN	Cross-sectional	*n* = 8115 (15–33 years)	Online survey	Decrease	Fair
70	Zhu et al., 2021 [18]	Hong Kong	QUAN	Cross-sectional	*n* = 2863 (9–17 years)	In-person survey	Increase	Fair
Africa								
71	Abid et al., 2021 [93]	Tunisia	QUAN	Cross-sectional	*n* = 100 (5–12 years)	Online survey	Decrease	Fair
72	Dixon et al., 2020 [53]	Kenya	QUAL	Cross-sectional	*n* = 16 (14–18 years)	Case study (observations, participant journals)	Decrease	Poor
Oceania								
73	Elliott et al., 2021 [94]	Australia	QUAL	Cross-sectional	*n* = 18 (15–17 years)	Online interviews, focus groups	Decrease	Poor
74	Masi et al., 2020 [95]	Australia	QUAN	Cross-sectional	*n* = 302 (2–17 years)	Online survey	Decrease	Poor
75	Munasinghe et al., 2020 [96]	Australia	QUAN	Longitudinal	*n* = 582 (13–19 years)	App survey, EMA, smartphone sensors	Decrease	Fair
76	Nathan et al., 2021 [31]	Australia	QUAN	Longitudinal	*n* = 157 (5–9 years)	Online survey	Unorganized PA: increase Organized PA: decrease	Fair
77	Parker et al., 2021 [41]	Australia	QUAN	Cross-sectional	*n* = 963 (13–17 years)	Online survey	-	Fair
78	Reece et al., 2020 [97]	Australia	QUAN	Cross-sectional	*n* = 16,177 (4–18 years)	Online survey	Decrease	Poor
79	Sciberras et al., 2020 [54]	Australia	QUAN	Longitudinal	*n* = 213 (5–17 years)	Online survey	Decrease	Fair
Cross-continental								
80	Guan et al., 2020 [98]	15 countries	QUAN	Cross-sectional	n/a	Community mobility data (Google)	Decrease	Poor
81	López-Aymes et al., 2021 [55]	Spain, Latin America	MIXED	Cross-sectional	*n* = 234 (≥18 years)	Online survey, discourse analysis	-	Poor
82	López-Gil et al., 2021 [99]	Brazil, Spain	QUAN	Cross-sectional	*n* = 1099 (3–17 years)	Online survey	Decrease	Fair
83	Okely et al., 2021 [24]	14 countries	QUAN	Longitudinal	*n* = 948 (3–5 years)	Survey	No difference	Fair
84	Ruíz-Roso et al., 2020 [32]	Brazil, Chile, Colombia, Spain, Italy	QUAN	Cross-sectional	*n* = 726 (10–19 years)	Online survey	Decrease overall (increase in Italy, Spain, Colombia)	Fair
